# Inhibition of RAC1 GTPase sensitizes pancreatic cancer cells to γ-irradiation

**DOI:** 10.18632/oncotarget.2500

**Published:** 2014-10-21

**Authors:** Ying Yan, Ashley L. Hein, Asserewou Etekpo, Katrina M. Burchett, Chi Lin, Charles A. Enke, Surinder K. Batra, Kenneth H. Cowan, Michel M. Ouellette

**Affiliations:** ^1^ Department of Radiation Oncology, University of Nebraska Medical Center, Omaha, Nebraska, United States of America; ^2^ Eppley Institute for Research in Cancer and Allied Diseases, University of Nebraska Medical Center, Omaha, Nebraska, United States of America; ^3^ Department of Biochemistry and Molecular Biology, University of Nebraska Medical Center, Omaha, Nebraska, United States of America

**Keywords:** ATM/ATR, clonogenic survival, G2 checkpoint, irradiation, radiosensitivity

## Abstract

Radiation therapy is a staple treatment for pancreatic cancer. However, owing to the intrinsic radioresistance of pancreatic cancer cells, radiation therapy often fails to increase survival of pancreatic cancer patients. Radiation impedes cancer cells by inducing DNA damage, which can activate cell cycle checkpoints. Normal cells possess both a G1 and G2 checkpoint. However, cancer cells are often defective in G1 checkpoint due to mutations/alterations in key regulators of this checkpoint. Accordingly, our results show that normal pancreatic ductal cells respond to ionizing radiation (IR) with activation of both checkpoints whereas pancreatic cancer cells respond to IR with G2/M arrest only. Overexpression/hyperactivation of Rac1 GTPase is detected in the majority of pancreatic cancers. Rac1 plays important roles in survival and Ras-mediated transformation. Here, we show that Rac1 also plays a critical role in the response of pancreatic cancer cells to IR. Inhibition of Rac1 using specific inhibitor and dominant negative Rac1 mutant not only abrogates IR-induced G2 checkpoint activation, but also increases radiosensitivity of pancreatic cancer cells through induction of apoptosis. These results implicate Rac1 signaling in the survival of pancreatic cancer cells following IR, raising the possibility that this pathway contributes to the intrinsic radioresistance of pancreatic cancer.

## INTRODUCTION

Radiation therapy is a staple treatment for pancreatic cancer patients, especially for those with locally advanced or borderline resectable disease [1, 2]. However, for the vast majority of pancreatic cancer patients, radiation therapy still fails to significantly increase survival or improve quality of life [3]. This lack of efficacy is primarily due to the intrinsic radioresistance of pancreatic cancer cells, as only 12–40% of pancreatic tumors exhibit an objective response to radiation therapy [4–22]. Thus, there is an urgent need to develop strategies for increasing the efficacy of radiation therapy in pancreatic cancer patients.

The ionizing radiation (IR) delivered by radiation therapy impedes cancer cells mainly by the production of DNA damage. In response to IR-induced DNA damage, human cells will engage several protective mechanisms that promote DNA repair and survival [23]. Among these is the activation of cell cycle checkpoints that block cell cycle progression to allow time for DNA repair [24]. Depending on the phase of the cell cycle at which the damage is sensed, cells can activate either a G1 or G2 checkpoint, to respectively block the cell cycle at the G1/S or G2/M border [24]. In cells that possess dysfunctional cell cycle checkpoints, apoptosis can instead occur to eliminate the damaged cells [25]. Normal cells have both a G1 and G2 cell cycle checkpoint to maintain their genomic integrity [26]. However, most cancer cells lack a functional G1 checkpoint, due to mutations/alterations in key regulators of the G1 checkpoint (e.g. p53, p16, and Cdk4) [26, 27]. For this reason, cancer cells are much more reliant on the functionality of the G2 checkpoint for their survival after radiation therapy.

The G2 checkpoint is tightly controlled by the Cdc2/Cyclin B complex, whose activity is required for the G2/M transition of the cell cycle [28]. Previous studies identify the Y15 residue of Cdc2 as a critical site in G2 checkpoint response to IR. Phosphorylation of Cdc2-Y15 following IR results in Cdc2 inhibition, leading to cell cycle arrest at the G2/M border [29–31]. Cdc2-Y15 is phosphorylated by the Wee1 and Myt1 kinases and dephosphorylated by Cdc25 dual-specificity phosphatases [32–34].

In response to IR exposure, ATM and ATR kinases are rapidly activated through phosphorylation, which, in turn, leads to the phosphorylation/activation of their respective downstream targets, the Chk1 and Chk2 kinases. Chk1 and Chk2 phosphorylate the Cdc25 phosphatases, resulting in the subcellular sequestration, degradation and/or inhibition of Cdc25, which normally activate Cdc2/Cyclin B complex at the G2/M boundary [35].

Cell cycle transition from G2 to mitotic phase requires histone H3 phosphorylation, which is associated with chromosome condensation prior to cell division [36]. Since both G2 and mitotic cells contain 4N-DNA content and are undistinguishable from each other by DNA content analysis, H3-Ser10 phosphorylation is commonly used as a marker of mitotic cells within the 4N population [37]. Histone H3-Ser10 phosphorylation begins in late G2 on the pericentromeric chromatin. As cells progress through mitosis, this phosphorylation has spread to the remaining chromatin by the end of prophase [38, 39]. Thus, there is a gradual increase in H3-Ser10 phosphorylation from the beginning to the end of mitosis. In a wide range of exponentially growing cells, H3-Ser10 phosphorylation in mitotic cells can be detected by flow cytometry analysis [40, 41]. Upon G2 checkpoint activation, H3-Ser10 phosphorylation is inhibited due to blockage of the G2/M transition of the cell cycle [28, 40, 41].

Ras-related C3 botulinum toxin substrate 1 (Rac1) is a member of the Rho family of small guanosine triphosphatases (GTPases). Rac1 has been shown to play a critical role in cytoskeleton reorganization, cell migration and cell survival [42]. Rac1 exists in either an active GTP-bound state or inactive GDP-bound state [43]. The transition of Rac1 between these two states is regulated by its GEFs (Guanine nucleotide Exchange Factors) and GAPs (GTPase–activating proteins). While GEFs promote Rac1 activation by accelerating GDP/GTP exchange, GAPs terminate Rac1 activity by promoting GTP hydrolysis [43]. In its active state, Rac1 interacts with its effectors, thereby activating numerous downstream signaling pathways [44, 45]. Overexpression/hyperactivation of Rac1 has been detected in the great majority of pancreatic cancers [46, 47]. Rac1 and two of its GEFs, Tiam1 and Vav1, have been reported to be overexpressed in more than 70% of pancreatic cancers, and Vav1 overexpression has also been associated with poor prognosis in pancreatic cancer patients [46–49]. Rac1 signaling has been shown to promote cellular transformation and to protect cells from apoptosis [43, 49].

While Rac1 is primarily localized at the cell membrane, it is also detected in the nucleus and the amount of nuclear Rac1 is increased in the late G2 phase [50]. Rac1 has been reported to activate ERK1/2 signaling via p21-activated kinase 1 and 2, which phosphorylate Raf1 and MEK1 and facilitates the formation of the Raf/MEK/ERK complex [51–53]. A role for Rac1 in the activation of PI3K/AKT pro-survival signaling has also been reported [54, 55] and Rac1 is necessary for the activation of AKT by UV and sphingosine 1-phosphate [56, 57]. Both AKT and ERK1/2 signaling pathways have been shown to promote cell survival after IR [23, 41, 58–62]. We recently reported a new function for Rac1 in the regulation of breast cancer cells' response to IR [63]. Our results revealed that Rac1 is rapidly activated in breast cancer cells after IR and that this activation is required for the activation of the G2 checkpoint response by IR and for cell survival following IR [63]. In the present study, we have investigated the role of Rac1 in the response of human pancreatic cancer cells to IR. Results in this report demonstrate that the inhibition of Rac1 sensitizes human pancreatic cancer cells to IR by a mechanism that involves G2 checkpoint abrogation and apoptosis induction.

## RESULTS

### IR exposure induces G2/M arrest and Cdc2 inhibition in pancreatic cancer cells

**Figure 1:**
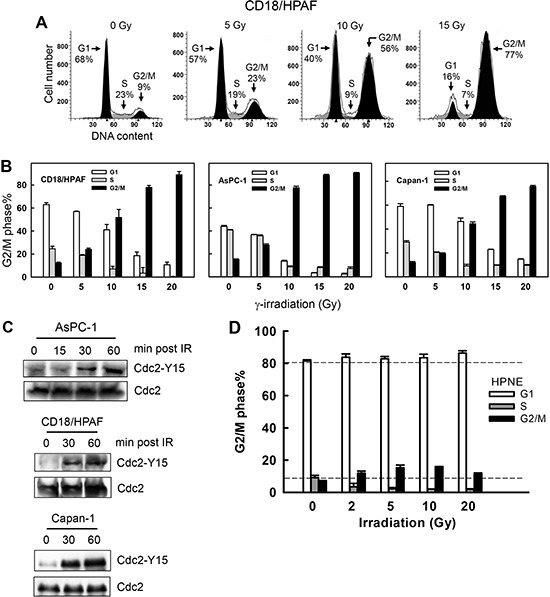
IR induces G2/M cell cycle arrest and Cdc2-Y15 phosphorylation in pancreatic cancer cells **(A)** Log-phase growing CD18/HPAF cells were exposed to increasing doses of IR, incubated for 24 h and analyzed for DNA content by FACS. Number of cells in G1, S and G2/M phases of the cell cycle are indicated. **(B)** Indicated pancreatic cancer cells were exposed to IR at the dose indicated, incubated for 24 h and analyzed for DNA content. Results depict the percentage of cells in G1 (white bars), S (gray bars) and G2/M (black bars) phases of the cell cycle and represent the mean±S.D. of two sets of experiments done in duplicates. **(C)** AsPC-1, CD18/HPAF and Capan-1 cells were exposed to 10 Gy IR, incubated for the indicated times and analyzed for Cdc2-Y15 phosphorylation as described in *MATERIALS AND METHODS*. As a control, levels of Cdc2 protein in cell lysates were assessed. **(D)** Normal human pancreatic ductal cells (HPNE) were exposed to IR at the doses indicated, incubated for 24 h and analyzed for DNA content by flow cytometry. The result depicts the percent cells in G1, S and G2/M phases of the cell cycle and is shown as the mean±S.D. of duplicate cell samples from two separate experiments.

To determine the response of pancreatic cancer cells to IR, exponentially growing pancreatic cancer cells were exposed to IR at the indicated doses and analyzed for DNA content by fluorescence-activated cell sorting (FACS) at 24 h following IR. As shown in Fig. 1A, IR exposure of CD-18/HPAF cells resulted in a marked increase in the amount of 4*N*-DNA content cells, indicative of G2/M phases of the cell cycle [28], and concomitant decreases in the amount of cells in G1 and S phases. Similarly, IR exposure of AsPC-1 and Capan-1 pancreatic cancer cells also resulted in a dose-dependent accumulation of G2/M phase cells, which was also associated with concomitant decreases in the amount of cells at G1 and S phases (Fig. 1B). These results indicate that these pancreatic cancer cells respond to IR exposure with a G2/M cell cycle arrest. This observation is consistent with the previous finding that most cancer cells possess a functional G2 checkpoint but are defective in G1 checkpoint [26, 27].

The G2 checkpoint activation requires inhibition of Cdc2, whose activity is required for G2/M transition of the cell cycle [28]. We next assessed changes in Cdc2-Y15 phosphorylation, indicative of Cdc2 inhibition, following IR exposure of pancreatic cancer cells. As shown in Fig. 1C, IR exposure resulted in a time-dependent increase in Cdc2-Y15 phosphorylation in AsPC-1, CD18/HPAF and Capan-1 pancreatic cancer cells, with the initial increase observed within 30 min following IR.

We also tested the response of normal human pancreatic ductal cells (HPNE) to IR. HPNE is a line of primary human pancreatic ductal cells immortalized with the catalytic subunit of human telomerase (hTERT) [64]. As shown in Fig. 1D, the majority of log-phase growing HPNE cells possessed 2N-DNA content, indicative of cells in G1 phase [65]. Following IR, cells in S phase were depleted as the amount of cells in both G1 and G2/M phases increased (Fig. 1D). This result indicates there were activations of both G1 and G2 checkpoints in HPNE cells following IR. Taken together, these results reveal a fundamental difference in cell cycle response to IR between normal and cancer cells. The normal pancreatic ductal cells have a G1 checkpoint response to IR that their cancer counterparts have lost.

### Rac1 is overexpressed in pancreatic cancer cells

**Figure 2:**
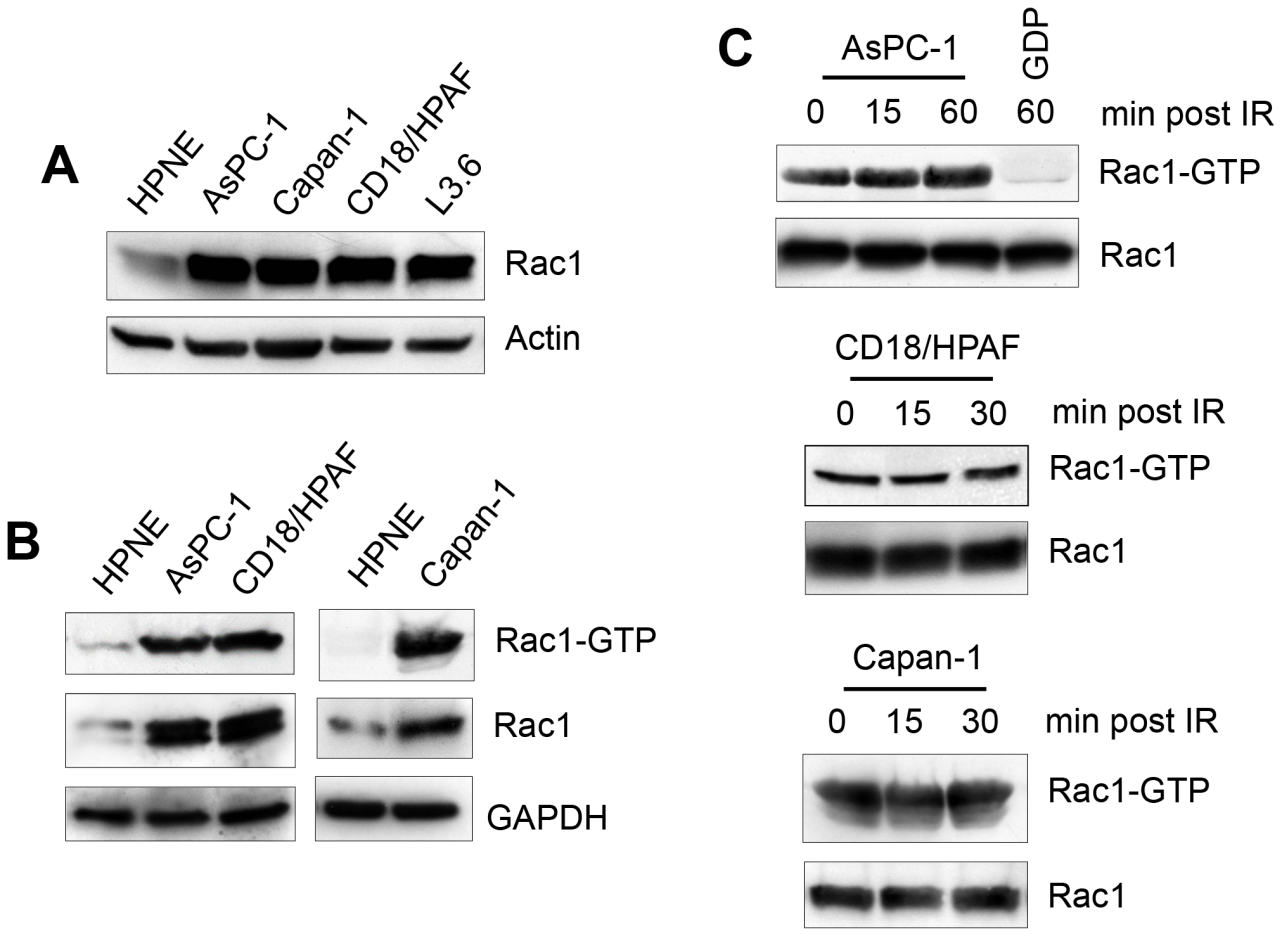
Rac1 is overexpressed in pancreatic cancer cells **(A)** Normal pancreatic ductal cells (HPNE) and pancreatic cancer cells (AsPC-1, Capan-1, CD18/HPAF and L3.6pl) were assessed for Rac1 protein expression by immunoblotting. **(B)** Indicated cells were analyzed for Rac1 activity (*Rac1-GTP*) as described in *MATERIALS AND METHODS*. As controls, protein levels of Rac1 (*Rac1*) and GAPDH (*GAPDH*) in cell lysates were measured. (C) AsPC-1, CD18/HPAF and Capan-1 cells were treated with 10 Gy IR and incubated for the indicated times and analyzed for the activity and level of Rac1.

Overexpression/hyperactivation of Rac1 in pancreatic cancer cells has been reported and Rac1 plays an important role in cell survival and transformation [47, 56, 66, 67]. To examine the role of Rac1 in the cellular response to IR, we analyzed Rac1 protein expression in HPNE and pancreatic cancer cells. As shown in Fig. 2A, the pancreatic cancer cells expressed much higher levels of Rac1 than HPNE primary human pancreatic ductal cells. Consistently, Rac1 activity assay demonstrated an association between Rac1 protein level and Rac1 activity in these cells, showing that much higher Rac1 activities were detected in AsPC-1, CD18/HPAF and Capan-1 pancreatic cancer cells compared to HPNE cells (Fig. 2B). We also assessed the pancreatic cancer cells for changes in Rac1 level and activity following IR. As shown in Fig. 2C, no noticeable change in Rac1 activity was detected after IR exposure of the cancer cells. These results indicate there is a marked increase in Rac1 level and activity in the pancreatic cancer cells relative to primary pancreatic ductal cells. In addition, this high level of Rac1 activity in the pancreatic cancer cells was unaffected by IR.

### Rac1 activity is necessary for IR-induced G2/M cell cycle arrest

**Figure 3:**
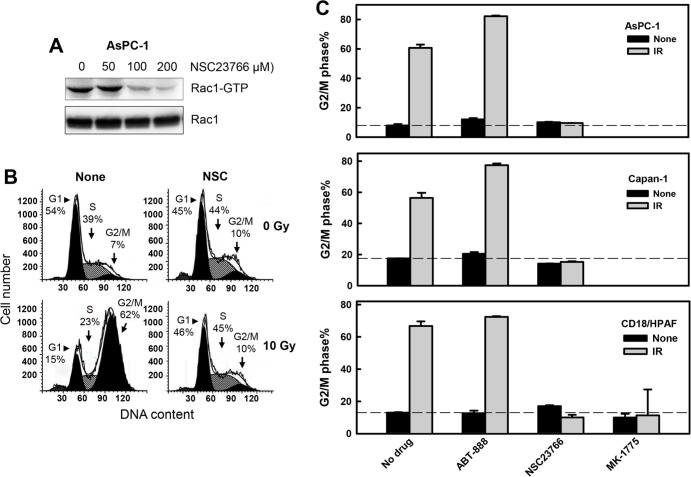

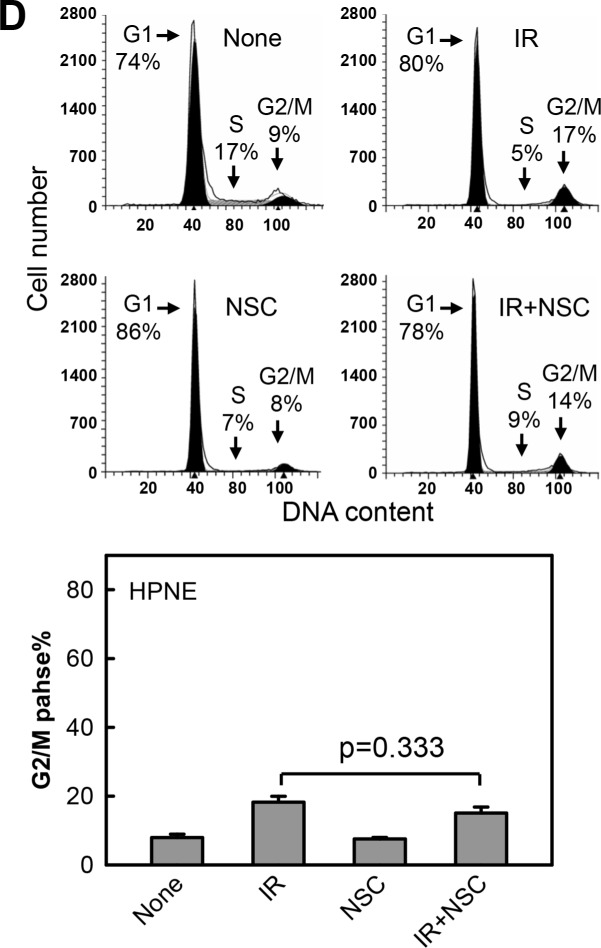
Rac1 inhibition abrogates IR-induced G2/M cell cycle arrest in pancreatic cancer cells **(A)** AsPC-1 cells were incubated for 1 h in the presence of NSC23766 at the indicated doses and analyzed for activity and level of Rac1. **(B)** AsPC-1 cells were incubated with 100 μM NSC23766 for 1 h, exposed to 10 Gy IR and incubated for 3 h post IR. The cells were washed, incubated in the absence of NSC23766 for 24 h and analyzed for DNA content by FACS. Number of cells in G1, S and G2/M phase of the cell cycle are indicated. **(C)** Indicated pancreatic cells were incubated for 1 h with ABT-888 (10 μM), NSC23766 (100 μM) and MK-1775 (3 μM), and exposed to 10 Gy IR. The cells were incubated for 3 h following IR, washed, incubated in the absence of drug for 24 h and analyzed for DNA content by FACS. Results depict the percentage of cells with 4*N*-DNA content (G2/M phase) and represent the mean±S.D. of two sets of experiments done in duplicates. **(D)** Upper panel: HPNE cells were incubated in the presence or absence of 100 μM NSC23766 for 1 h, exposed to 10 Gy IR, incubated for 24 h and analyzed for DNA content. Number of cells in G1, S and G2/M phase of the cell cycle are indicated. Lower panel: The result depicts the percentage of cells with *4N*-DNA content (G2/M phase) and is shown as mean±S.D. of duplicate samples from two separate experiments.

Using the Rac1 specific inhibitor NSC23766 [68], we examined the effect of Rac1 on IR-induced G2/M arrest in pancreatic cancer cells. For these experiments, pancreatic cancer cells (AsPC-1, Capan-1 and CD18/HPAF) were incubated with increasing doses of NSC23766 and exposed to IR. As shown in Fig. 3A, incubation of AsPC-1 cells with NSC23766 at 100 and 200 μM resulted in 62% and 83% inhibition of Rac1 activity, respectively, compared to control untreated cells (*Rac1-GTP*). As shown in Figs. 3B and 3C, incubation of AsPC-1 cells in the presence of 100 μM NSC23766 resulted in a marked attenuation in the induction of G2/M arrest following IR. In contrast, incubation with NSC23766 alone in the absence of IR only had subtle, if any, effect on the percentage of *4N*-DNA content cells relative to log-phase growing cells (Fig. 3B, *NSC*). Incubation of AsPC-1 cells with NSC23766 also resulted in a slight decrease in the amount of G1 phase cells and a minor increase, if any, in the amount of S phase cells (Fig. 3B, *NSC*). Furthermore, incubation with 100 μM NSC23766 also abrogated the IR-induced G2/M arrest in Capan-1 and CD18/HPAF pancreatic cancer cells (Fig. 3C).

As additional controls, the indicated pancreatic cancer cells were treated with ABT-888, a poly(ADP-ribose) polymerase 1 and 2 (PARP1/2) inhibitor [69], or MK-1775, a Wee1 kinase inhibitor [70] and then exposed to IR. While PARP1/2 plays an essential role in the repair of single-stranded DNA breaks, Wee1 inhibits Cdc2 kinase by phosphorylation of the Y15 residue of Cdc2 [71, 72]. The two drugs were used at concentrations sufficient to respectively inhibit protein poly(ADP-ribosyl)ation and Cdc2-Y15 phosphorylation as shown in Supplementary Fig. 1, as well as described previously [69, 70]. As shown in Fig. 3C, incubation with MK-1775 completely abrogated the IR-induced G2/M arrest in the pancreatic cancer cells, whereas incubation with ABT-888 did not block the induction of G2/M arrest after IR.

As an additional control, we also investigated the effects of Rac1 inhibition on the response of HPNE primary pancreatic ductal cells to IR. As shown in Fig. 3D, Rac1 inhibition by NSC23766 had little effects on the IR-induced cell cycle response of HPNE cells.

**Figure 4:**
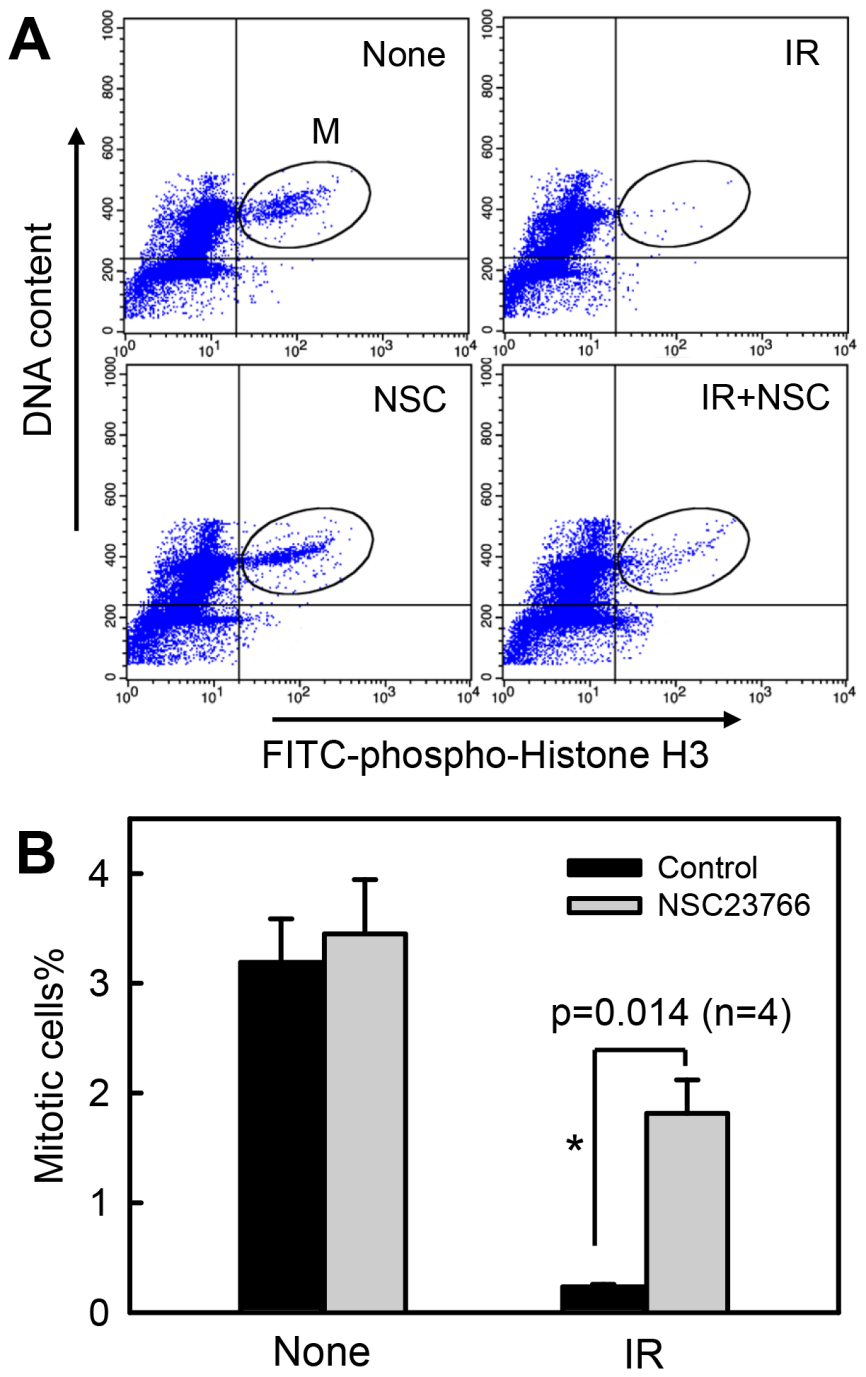
Rac1 inhibition abrogates IR-induced G2/M checkpoint activation CD18/HPAF cells were incubated for 1 h in the presence or absence of 100 μM NSC23766, treated with/without 10 Gy IR. After 2 h incubation following IR, the cells were analyzed by FACS for mitotic cells, which contain both *4N*-DNA content and Histone H3-Ser10 phosphorylation [37]. **(A)** The histograms shown are representative FACS analyses for mitotic cells in samples treated with/without IR in the presence or absence of NSC23766. The location of mitotic cells in each sample is indicated (*M*). **(B)** The bar graph depicts the percentage of mitotic cells and is shown as mean±S.D. of duplicate samples from two set of experiments. *, significant difference from cells exposed to IR in the absence of NSC23766.

Using histone-H3 phosphorylation as a marker of mitotic cells [73], we examined the effect of Rac1 on the proportion of cells in mitosis following IR exposure. As shown in Fig. 4, IR exposure resulted in a rapid decrease in the proportion of mitotic cells in CD18/HPAF cells. At 2 h post IR, there was an approximately 90% decrease in mitotic cells relative to non-irradiated control cells (Fig. 4A: *IR vs. None*; Fig. 4B: black bars). In contrast, incubation of cells with NSC23766 blocked the effect of IR, resulting in a significant increase in the proportion of mitotic cells in irradiated cells compared to the control irradiated cells incubated in the absence of NSC23766 (Fig. 4A: *IR+NSC vs. IR*; Fig. 4B: IR). Incubation of cells with NSC23766 alone resulted in only a slight increase in the amount of mitotic cells compared to the control untreated cells (Fig. 4A: *NSC vs. None*; Fig. 4B: None).

### Inhibition of Rac1 abolishes IR-induced ATM and ATR signaling activation

**Figure 5:**
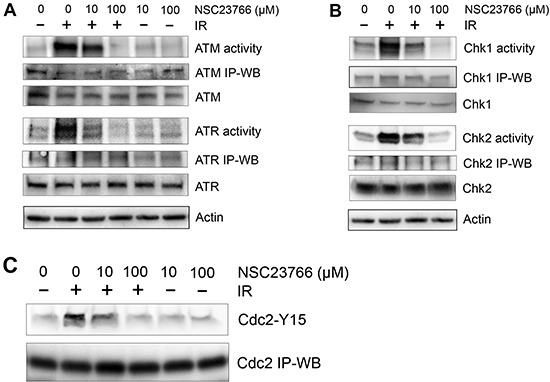
Rac1 inhibition abolishes IR-induced activation of both ATM and ATR signaling pathways CD18/HPAF cells were treated with/without 10 Gy IR in the presence of NSC23766 at the indicated doses and incubated for 1 h at 37^o^C. **(A)** To assess ATR and ATM kinase activities, ATR and ATM were immunoprecipitated from the cell lysates using anti-ATR (N-19) and anti-ATM (2C1) antibodies respectively and assayed for relative kinase activity using recombinant p53 protein as substrate. **(B)** To measure Chk1 and Chk2 activity, Chk1 and Chk2 were immunoprecipitated from the cell lysates using anti-Chk1 (G-4) and anti-Chk2 (B-4) antibodies respectively and assayed for relative kinase activity using recombinant Cdc25C protein as substrate. As controls, protein levels of ATR, ATM, Chk1 and Chk2 in the immunoprecipitates (*IP-WB*) as well as in the cell lysates (*WB*) were assessed by immunoblotting. **(C)** Cdc2 was immunoprecipiated from the cell lysates using anti-Cdc2 (17) antibody and analyzed for Cdc2-Y15 phosphorylation and Cdc2 protein by immunoblotting.

To investigate the mechanisms involved in the regulation of the IR-induced G2/M checkpoint response by Rac1, we examined the effect of Rac1 on the activation of ATM and ATR signaling after IR. As shown in Fig. 5A, pre-incubation of CD18/HPAF cells with NSC23766 resulted in a dose-dependent diminution of IR-induced activation of ATM and ATR kinase activities. A complete inhibition of both IR-induced ATM and ATR activities was achieved in cells incubated with 100 μM NSC23766 and exposed to IR.

To confirm the effect of Rac1 inhibition on IR-induced activation of ATM and ATR kinases, we analyzed Chk1 and Chk2 activities in CD18/HPAF cells following IR exposure with or without the presence of NSC23766. As shown in Fig. 5B, while IR induced activation of both Chk1 and Chk2 in CD18/HPAF cells, the effect was dose-dependently blocked by the inhibition of Rac1. Consistent with the effect of NSC23766 on the IR-induced ATM and ATR, presence of 100 μM NSC23766 completely abrogated the IR-induced Chk1 and Chk2 activation in CD18/HPAF cells (Fig. 5B, *Chk1 activity* and *Chk2 activity*).

Activity of the Cdc2/Cyclin B complex is essential for the cells to progress from G2 to M phase of the cell cycle [74]. Activation of ATM and ATR signaling following IR leads to the induction of Cdc2-Y15 phosphorylation that inhibits Cdc2 activity [75]. We therefore examined the effect of Rac1 inhibition on the IR-induced Cdc2-Y15 phosphorylation in CD18/HPAF cells. As shown in Fig. 5C, IR-induced Cdc2-Y15 phosphorylation was inhibited dose-dependently by the presence of NSC23766. Consistently, incubation with 100 μM NSC23766, a dose that abrogated the activation of ATM/Chk2 and ATR/Chk1 following IR, also inhibited the induction of Cdc2-Y15 phosphorylation in CD18/HPAF cells after IR (Fig. 5C).

Collectively, results in Fig. 5 indicate that Rac1 inhibition by NSC23766 abolished the IR-induced activation of ATM and ATR signaling pathways, which play key roles in the regulation of G2 checkpoint response.

### Ectopic expression of dominant negative N17Rac1 mutant blocks G2/M checkpoint activation following IR

**Figure 6:**
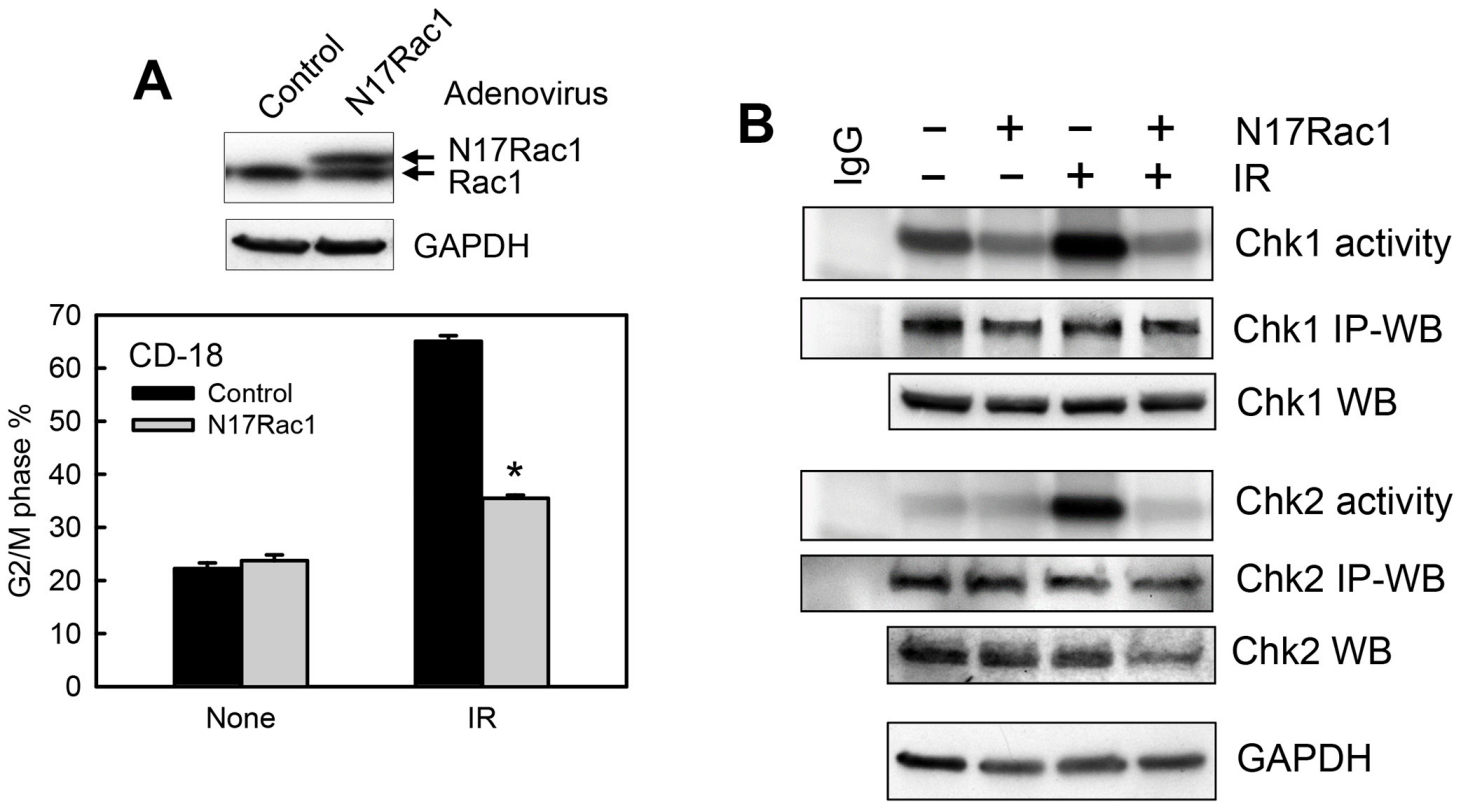
Ectopic expression of N17Rac1 dominant mutant diminishes IR-induced G2/M checkpoint activation **(A)** Upper panel: CD18/HPAF cells were transduced with adenoviral vector expressing N17Rac1 or control vector for 24 h and analyzed for Rac1 and GAPDH by immunoblotting. Lower panel: The transduced cells were treated with or without IR, incubated for 24 h and analyzed for DNA content by FACS. The result depicts the percentage of cells with *4N*-DNA content and is shown as mean±S.D. of duplicate samples from two separate experiments. *, *p*< 0.001 (n=4), significant difference from the control vector transduced cells exposed to IR. **(B)** The transduced cells were treated with or without IR, incubated for 1 h and analyzed for the activities of Chk1 and Chk2 by kinase assay. As controls, protein levels of Chk1 and Chk2 in the immunoprecipitates (*IP-WB*) as well as in the cell lysates (*WB*) were assessed by immunoblotting. GAPDH protein in the cell lysates was assessed by Western blotting as a protein loading control.

Using an adenoviral vector expressing N17Rac1, a dominant negative mutant of Rac1 [76], we verified the effect of Rac1 inhibition on the IR-induced G2/M checkpoint response in CD18/HPAF cells. For these studies, the cells were transduced with adenoviral vector expressing N17Rac1 (Ad.N17Rac1) or control empty vector (Ad.Control). As shown in Fig. 6A (upper panel), immunoblotting analysis detected the ectopically expressed N17Rac1, which migrates slightly slower than the endogenous wild-type Rac1. We next examined the effect of N17Rac1 mutant on IR-induced G2/M arrest. As shown in Fig. 6A (lower panel), DNA-content analyses revealed a marked induction in IR-induced G2/M arrest in the control vector transduced CD18/HPAF cells and that this induction was blocked significantly by the expression of N17Rac1 (*p*<0.001, n=4).

We next examined the effect of N17Rac1 expression on the activation of Chk1 and Chk2 following IR. As shown in Fig. 6B, while control vector-transduced CD18/HPAF cells showed a noticeable activation of both Chk1 and Chk2 kinases after IR, N17Rac1-transduced cells exhibited a marked diminution in the activation of Chk1 and Chk2 following IR compared to the control vector-transduced cells (*Chk1 activity* and *Chk2 activity*). In addition, N17Rac1 expression also resulted in a slight decrease in basal Chk1 activity in the un-irradiated cells (Fig. 6B). Transduction of CD18/HPAF cells with control vector had no noticeable effect on IR-induced activation of Chk1 and Chk2 compared to un-transduced cells (data not shown).

### Inhibition of Rac1 sensitizes pancreatic cancer cells to IR exposure

**Figure 7:**
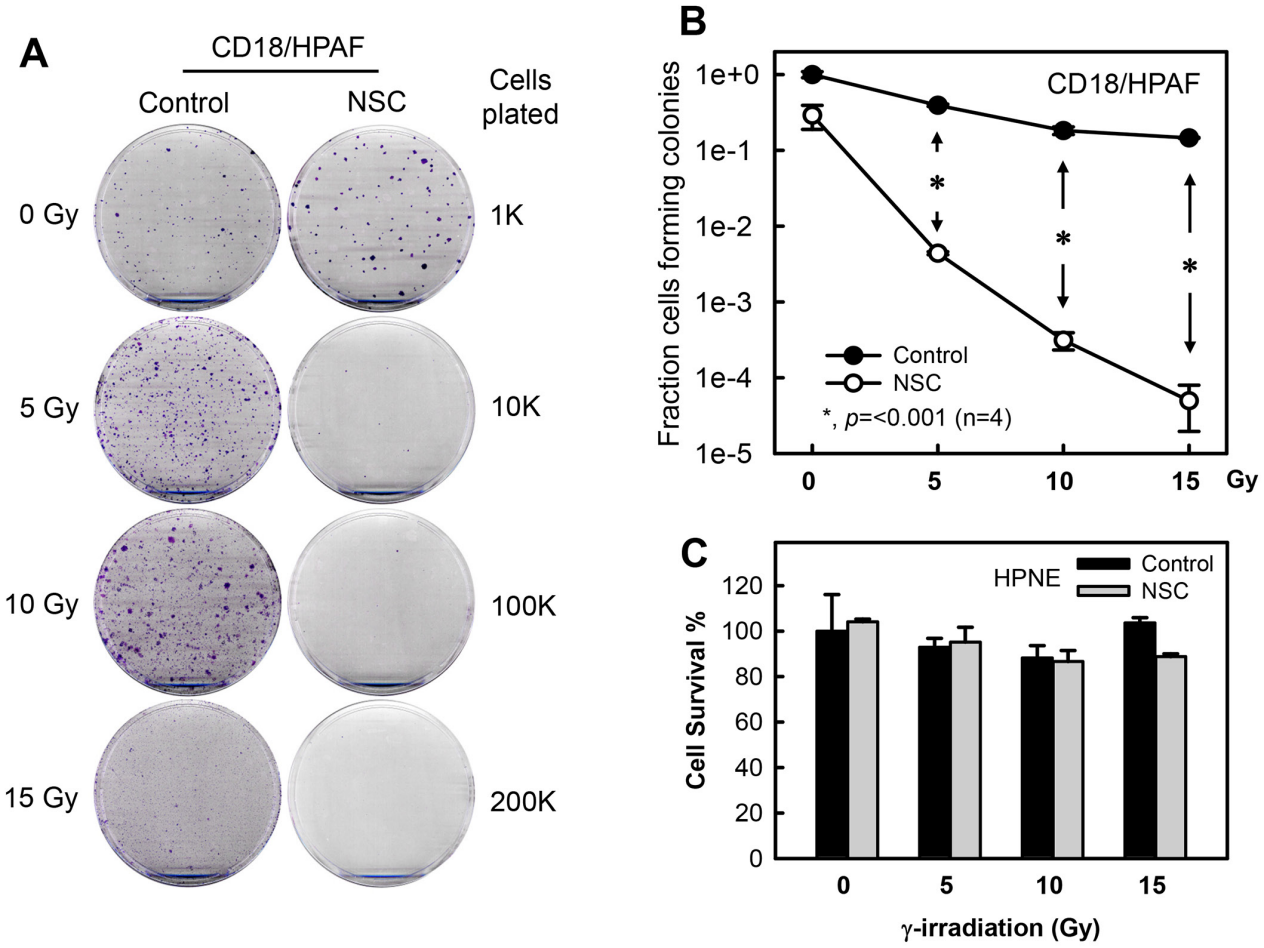

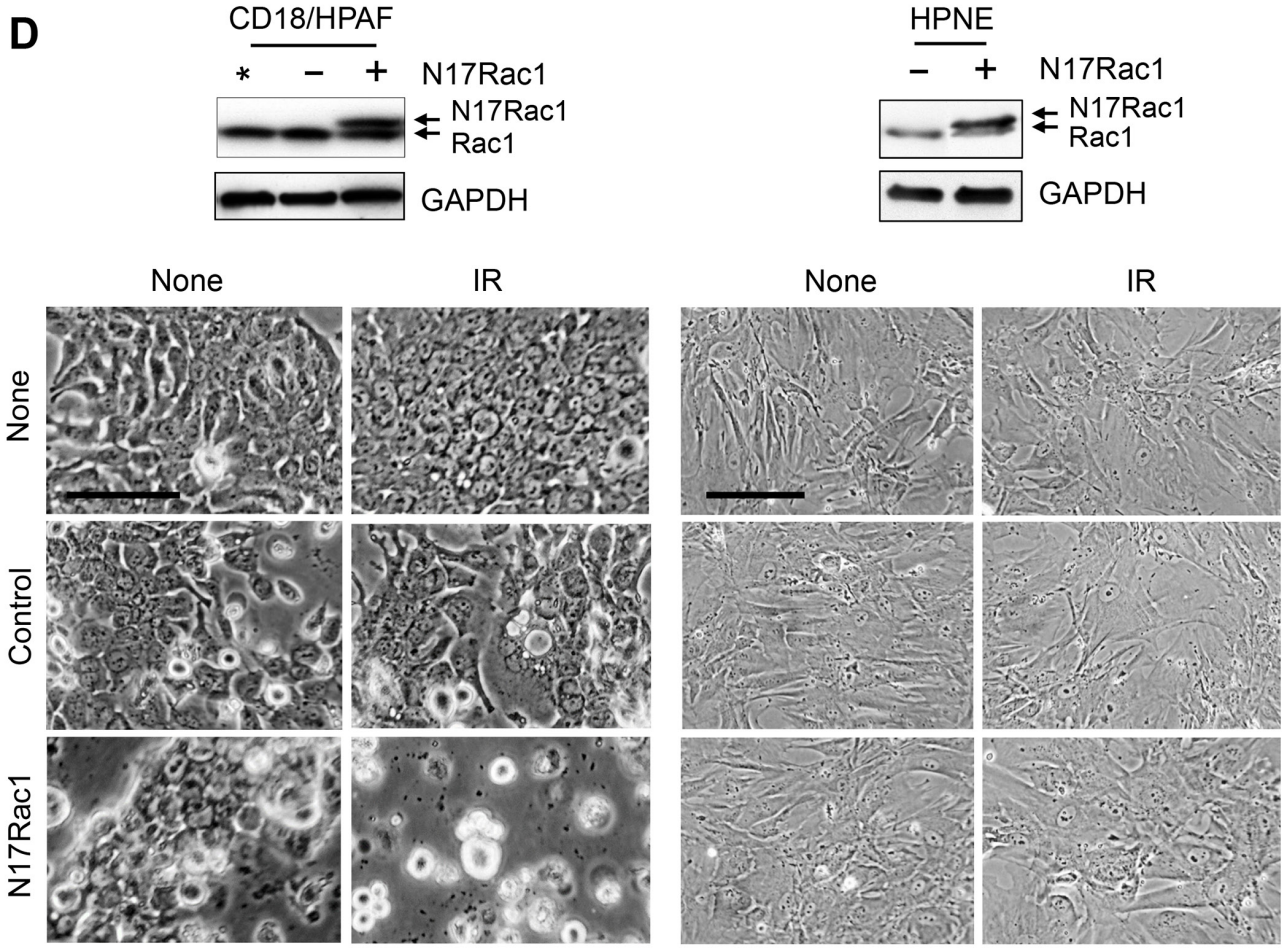
Inhibition of Rac1 abrogates clonogenic survival of irradiated pancreatic cancer cells (A) CD18/HPAF cells were exposed to increasing doses of IR in the presence or absence of 100 μM NSC23766 and incubated for 3 h. The cells were washed, incubated in regular medium for 14 days and assessed for numbers of colonies [63]. Representative sample dishes from the clonogenic assay are shown. (B) Number of colonies in the resulting samples (CD18/HPAF) was quantified using the ImageJ analytical program and the results are shown as mean±S.D. of two set of experiments done in duplicates. *, *p*=<0.001 (n=4), significant difference between the cells exposed to IR in the absence of NSC23766 and the cells exposed to IR in the presence of NSC23766. (C) HPNE cells were treated as described in (A). Cell survival in the resulting cell samples was quantified using the ImageJ analytical program and the results are shown as mean±S.D. of two set of experiments done in duplicates. **(D)** CD18/HPAF and HPNE cells were transduced with Ad.N17Rac1 (*+*) or Ad.Control (−) for 24 h. Upper panels: Western blot analysis of the indicated samples for Rac1 and GAPDH. *, un-transduced CD18/HPAF control cells. Lower panels: cells were treated with or without 10 Gy IR and incubated for additional 48 h. Cells were photographed using phase-contrast optics. Scale bars represent 100 μm.

Results in Figs. 1–6 showed that the IR-induced G2/M checkpoint activation in human pancreatic cancer cells was abrogated by the Rac1 specific inhibitor NSC23766 and by expression of the N17Rac1 mutant. We next examined the effect of Rac1 inhibition on cell survival after IR using a clonogenic assay. As shown in Figs. 7A and 7B, while IR exposure alone resulted in only a small decrease in clonogenic survival of CD18/HPAF cells, IR exposure in the presence of NSC23766 resulted in a striking decrease in clonogenic survival of these cells. In the presence of NSC23766, cell viability after 5, 10 and 15 Gy of IR was respectively decreased by 2, 3 and 4 orders of magnitude compared to their corresponding irradiated controls (Fig. 7B). In contrast, treatment of cells with NSC23766 alone in the absence of IR only resulted in a subtle decrease, if any, in cell survival relative to the untreated control cells. However, the NSC23766 treated cells appeared to form larger colonies compared to the untreated control cells (Fig. 7A, *0 Gy*: *Control vs. NSC*). We also tested the effect of Rac1 inhibition on the viability of irradiated HPNE normal cells, which express a much lower level of Rac1 protein relative to CD18/HPAF pancreatic cancer cells (Fig. 2). As shown in Fig. 7C, inhibition of Rac1 by NSC23766 had little effect on the viability of HPNE cells following IR.

To verify the effect of Rac1 inhibition on cell survival following IR, we transduced CD18/HPAF pancreatic cancer cells and HPNE normal cells with Ad.N17Rac1 or Ad.Control viruses and exposed the cells to IR. Results in Fig. 7D (upper panels) confirmed the presence of ectopic N17Rac1 expression in the Ad.N17rac1-transduced CD18/HPAF and HPNE cells. As shown in Fig. 7D (lower left panel), expression of N17Rac1 in the absence of IR resulted in visible morphological changes in the CD18/HPAF cells compared to the control-infected cells. At 2 days following IR, N17Rac1-expressing CD18/HPAF cells had rounded up and had detached from the culture dish (Fig. 7D, lower left panel, *N17Rac1* + *IR*), whereas Ad.Control-infected CD18/HPAF cells remained attached and showed little change in cell morphology compared to the unirradiated Ad.Control-infected cells (Fig. 7D, lower left panel, *Control* + *IR vs. Control* + *None*). In contrast, in HPNE cells, neither N17Rac1 expression nor IR produced any noticeable changes in cell morphology (Fig. 7D, lower right panel). In both cell lines, control viral infection had little effect on cell morphology relative to their respective uninfected cells (Fig. 7D, lower panels: *Control vs. None*).

In summary, the results of these studies indicate that the inhibition of Rac1, either by NSC23766 or expression of N17Rac1, augments the sensitivity of CD18/HPAF pancreatic cancer cells to IR, whereas it has little effect on the sensitivity of HPNE normal cells to IR.

### Rac1 inhibition results in apoptosis induction in pancreatic cancer cells exposed to IR

**Figure 8:**
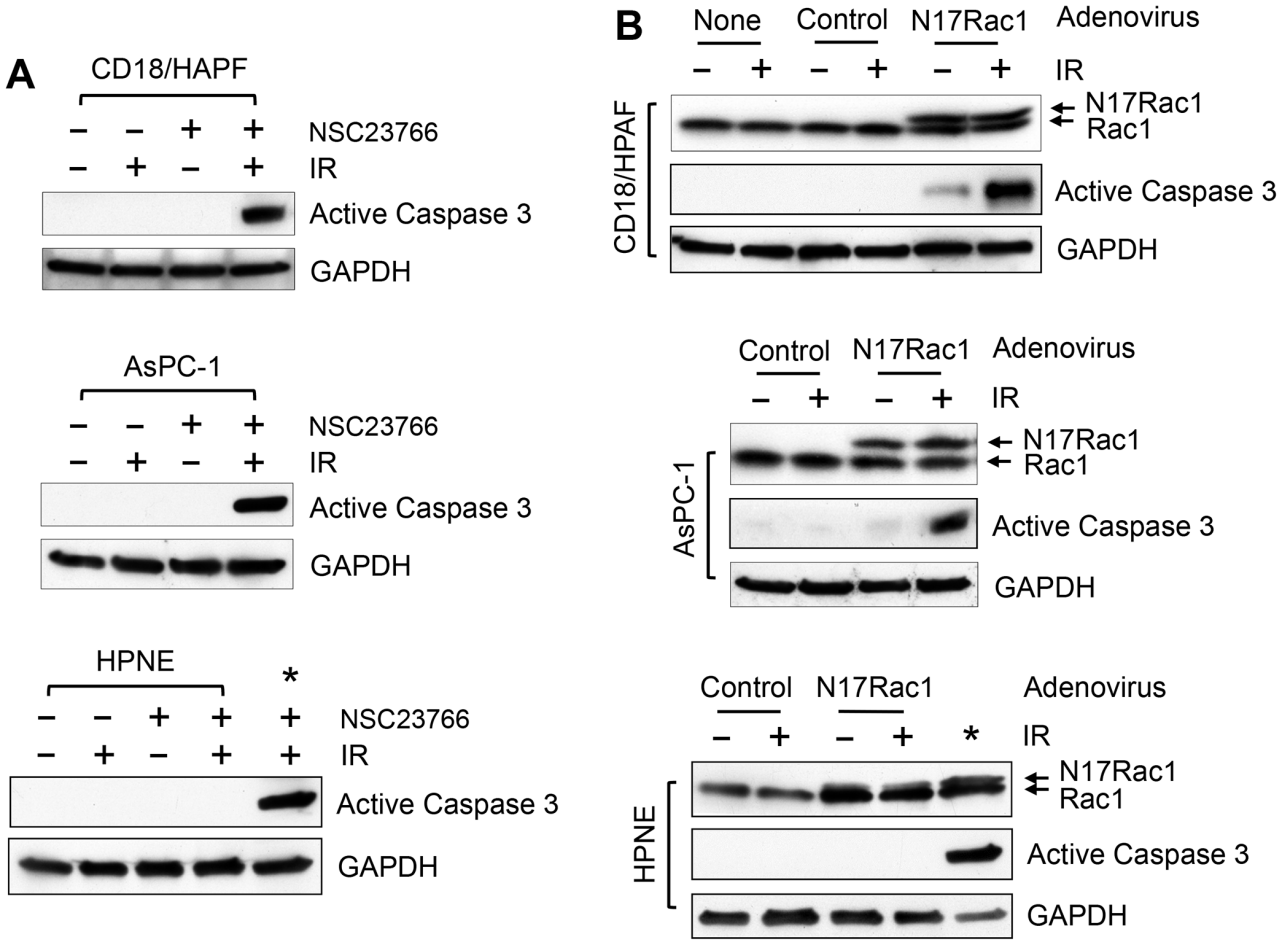
Inhibition of Rac1 induces Caspase 3 activation in pancreatic cancer cells following IR **(A)** The indicated cells were treated with/without 10 Gy IR in the presence or absence of 100 μM NSC23766 and incubated for 2 days. The cells were analyzed by immunoblotting for levels of activated Caspase 3 (p20) and GAPDH. *, positive control for caspase 3 activation: AsPC-1 cells treated with NSC23766 and IR. **(B)** The indicated cells were infected with Ad.N17Rac1 or Ad.Control for 24 h and exposed to 10 Gy IR or left non-irradiated. Following 24 h incubation, cells were examined by immunoblotting for levels of Rac1, activated Caspase 3 (p20) and GAPDH. *, positive control for caspase 3 activation: CD18/HPAF cells were transduced with Ad.N17Rac1 for 24 h, exposed to 10 Gy and incubated for 24 h.

To investigate the possible mechanisms involved in the increase in radiation sensitivity in pancreatic cancer cells by Rac1 inhibition, we assessed the treated cells for markers of apoptosis induction. It has been previously demonstrated that the activation of caspase 3, a hallmark of apoptosis induction, occurs during the execution phase of apoptosis [77]. As shown in Fig. 8A (upper and middle panels), at 2 days after IR, immunoblotting detected the presence of activated caspase 3 (p20), indicative of apoptosis induction, in both the AsPC-1 and CD18/HPAF cells treated with NSC23766. In contrast, no evidence of caspase 3 activation was detected in the cells treated with either NSC23766 alone or IR alone (Fig. 8A, upper and middle panels). For a comparison, we also assessed caspase 3 activation in HPNE normal cells treated with IR and/or NSC23766. As shown in Fig. 8A (lower panel), no evidence of caspase 3 activation was detected in any of the HPNE samples, whether treated with IR and/or NSC23766. In contrast, the activated caspase 3 was readily detected in the positive control, AsPC-1 cells treated with both NSC23766 and IR.

To verify the effect of Rac1 inhibition on caspase 3 activation following IR, CD18/HPAF, AsPC-1 and HPNE cells were transduced with N17Rac1 or control viral vector and exposed to IR. As shown in Fig. 8B, activation of caspase 3 was detected in both the CD18/HPAF and AsPC-1 cells transduced with N17Rac1 and exposed to IR, but not in the control viral vector infected cells exposed to IR. Expression of N17Rac1 by itself also resulted in a detectable but limited caspase 3 activation in CD18/HPAF cells (Fig. 8B, upper panel). But in AsPC-1 cells, N17Rac1 by itself did not lead to caspase 3 activation (Fig. 8B, middle panel). In contrast, ectopic N17Rac1 expression did not cause caspase 3 activation in HPNE cells, either with or without IR (Fig. 8B, bottom panel). Thus, the effect of N17Rac1 on the induction of apoptosis following IR appears to be cancer specific, as the pancreatic cancer cell lines were more susceptible to this effect than HPNE cells.

In summary, results of these studies indicate that the inhibition of Rac1 using either pharmacological inhibitor or dominant negative mutant promotes apoptosis induction after IR in pancreatic cancer cells. However, Rac1 inhibition has little effect on the survival of normal pancreatic ductal cells following IR.

### Rac1 inhibition abolishes IR-induced AKT activation in pancreatic cancer cells

**Figure 9:**
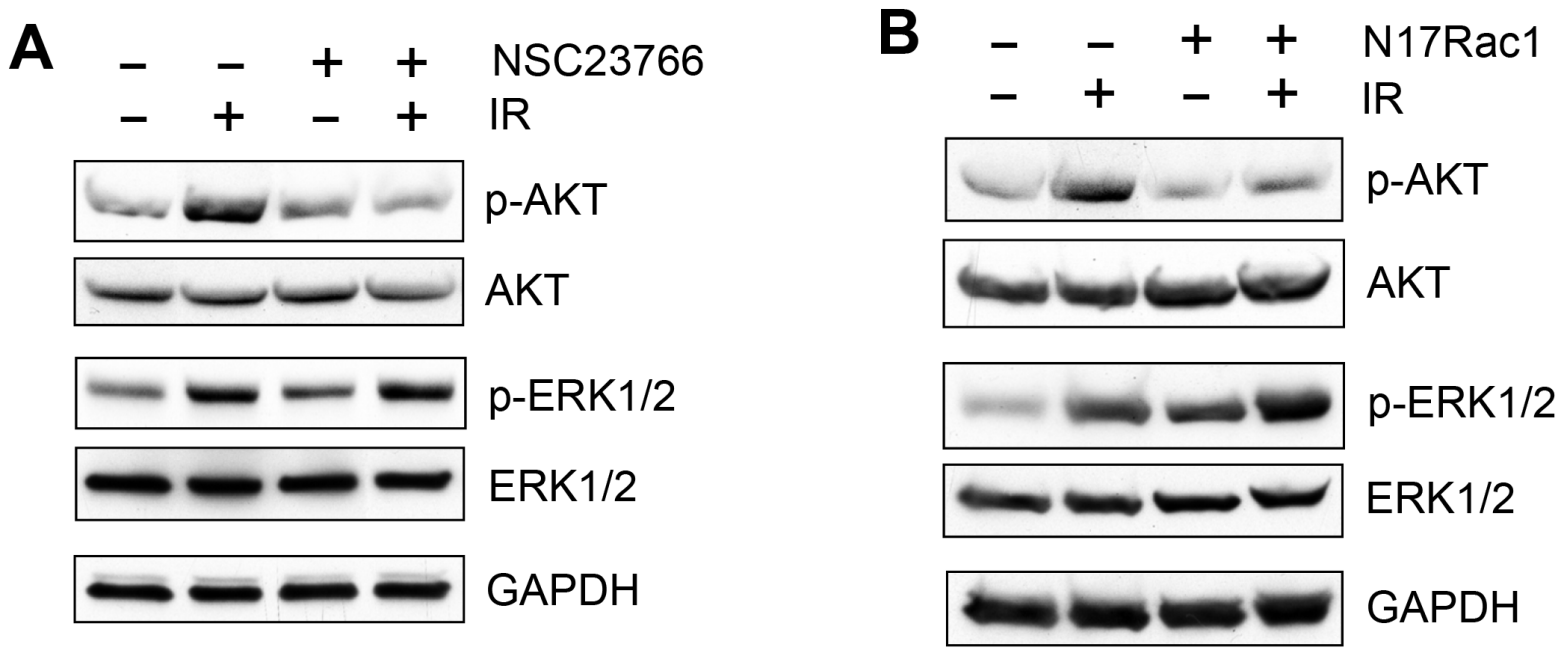
Effect of Rac1 inhibition on IR-induced AKT and ERK1/2 phosphorylation **(A)** In the presence or absence of 100 μM NSC23766, CD18/HPAF cells were treated with/without IR and analyzed for phosphorylation and level of AKT and ERK1/2 by immunoblotting. GAPDH was assessed as a protein loading control. **(B)** CD18/HPAF cell were infected with Ad.N17Rac1 or Ad.Control for 24 h and exposed to 10 Gy IR or un-irradiated. Following 1 h incubation post IR, the cells were examined for phosphorylation and level of AKT and ERK1/2. GAPDH was assessed as a protein loading control.

Both AKT and ERK1/2 signaling pathways have been shown to promote cell survival in response to radiation [23]. Since Rac1 has been shown to activate AKT and ERK1/2 in response to various stimuli [56, 57, 78, 79], we tested the effect of Rac1 inhibition on the IR induced activation of AKT and ERK1/2. As shown in Fig. 9A, while a marked increase in AKT phosphorylation (*pAKT*), indicative of AKT activation, was detected in CD18/HPAF cells following IR, this effect of IR was diminished in the cells incubated with Rac1 inhibitor NSC23766. In contrast, the IR-induced ERK1/2 phosphorylation, indicative of ERK1/2 activation, was unaffected by the incubation of CD18/HPAF cells with NSC23766 (Fig. 9A, *pERK1/2*). Treatment with IR and/or NSC23766 had no detectable effect on the overall levels of AKT and ERK1/2 proteins (Fig. 9A, *AKT* and *ERK1/2*).

The effect of Rac1 on IR-induced activation of AKT and ERK1/2 was also examined using N17Rac1 mutant. As shown in Fig. 9B, ectopic expression of N17Rac1 in CD18/HPAF cells resulted in a significant diminution of IR-induced AKT phosphorylation (*pAKT*), whereas it did not block the increase of ERK1/2 phosphorylation following IR (*pERK1/2*). This result is consistent with the effect of Rac1 inhibitor NSC23766, suggesting that Rac1 plays an essential role in the IR-induced AKT activation in CD18/HPAF pancreatic cancer cells whereas it has little effect on the IR-induced ERK1/2 activation in these cells.

## DISCUSSION

Rac1 is constitutively activated in the great majority of pancreatic cancers and contributes critically to the development and maintenance of pancreatic cancer [46, 47]. Rac1 and two of its GEFs, Tiam1 and Vav1, are overexpressed in more than 70% of pancreatic cancers [46–48]. We also observe in the present study a striking up-regulation of Rac1 level/activity in cancerous versus normal pancreatic cells (see Fig. 2). The Rac1 signaling pathway is required for transformation mediated by the Ras oncogene [80–83] and, in the mouse K-Ras^G12D^ knock-in model of pancreatic cancer, Rac1 is required for the development of tumors [84, 85]. The pathway promotes transformation, protects from apoptosis, and promotes motility and invasion [46, 48, 84, 86]. In this report, we provide evidence that the Rac1 pathway also plays an essential role in the response of pancreatic cancer cells to IR. Our results suggest that the hyperactivation of this pathway protects pancreatic cancer cells from the deleterious effects of radiotherapy.

We have recently identified the Rac1 signaling pathway as an important regulator of the response of breast cancer cells to IR [63]. In breast cancer cells, Rac1 is activated by IR and the inhibition of Rac1 abrogates G2 checkpoint activation and cell survival following IR. In the present report, we uncovered a similar role played by Rac1 in pancreatic cancer cells. Pancreatic cancer cells are notoriously resistant to the toxicity of radiation therapy. Nonetheless, inhibition of Rac1 in pancreatic cancer cells with a specific inhibitor or a dominant negative mutant of Rac1 is sufficient to abrogate the IR-induced G2 checkpoint activation, as evidenced by cell cycle analyses, histone H3 phosphorylation, and activity assessments of ATR/Chk1 and ATM/Chk2 kinases (see Fig. 3–6). The inhibition of Rac1 also abrogates the IR-induced AKT activation, which plays an important role in antagonizing apoptosis induction. The net effect of these alterations caused by Rac1 inhibition is a marked increase in radiosensitivity of pancreatic cancer cells, as demonstrated by caspase 3 activation, production of floating cells and the results of clonogenic survival assays (see Fig. 7–8). These results reveal an important role for Rac1 pathway in protecting pancreatic cancer cells from the cytotoxic effects of IR. The data raises the possibility that the intrinsic radioresistance of pancreatic cancer cells might be a consequence of the constitutive activation of the Rac1 pathway in this disease. Further studies will be needed to test this possibility and to decipher the mechanisms involved, as well as relative contributions of G2 checkpoint abrogation and AKT inhibition to the radiosensitizing activities of Rac1 inhibitors.

Activation of AKT and ERK1/2 signaling pathways following IR has been associated with cell survival after IR [87, 88]. It has also been shown that Rac1 is necessary for PI3K/AKT activation by lipopolysaccharides and MEK/ERK activation by 12-O-tetradecanoylphorbol-13-acetate [79, 89]. These reports initially led to our hypothesis that both AKT and ERK1/2 were downstream targets of Rac1 in the response of pancreatic cancer cells to IR. However, although IR induces activation of both AKT and ERK1/2 in CD-18/HPAF cells, inhibition of Rac1 abrogates only the AKT activation after IR but not the IR-induced ERK1/2 activation (see Fig. 9). These results suggest an involvement of AKT but not ERK1/2 in the survival of pancreatic cancer cells following IR. We will investigate the regulation of IR-induced AKT signaling by Rac1 in future studies.

A common pitfall of radiation therapy in pancreatic cancer patients is the proximity of critical structures, including healthy pancreas, surrounding blood vessels, and gastric epithelium. To be valuable in the clinic, an ideal radiosensitizer should selectively sensitize cancer cells and leave normal cells unaffected. To address this issue, we have compared the response of pancreatic cancer cells to IR and Rac1 inhibition with that of normal pancreatic ductal cells. Our results indicate that Rac1 inhibition only has little effects on the response of the normal cells to IR. Most significantly, survival of normal pancreatic ductal cells following IR is only marginally affected by the inhibition of Rac1, in stark contrast with the radiosensitization observed in the pancreatic cancer cell lines. The mechanisms responsible for the differential effects of Rac1 inhibitors are unknown. Two major differences between normal and cancer cells may play a role in this differential response to IR. First, there is a marked difference in Rac1 activity between the normal pancreatic ductal cells and pancreatic cancer cells (see Fig. 2). The high Rac1 activity in the pancreatic cancer cells may make these cells more dependent on Rac1 for survival. Second, most cancer cells have a defective G1 checkpoint made dysfunctional by mutations in regulators of the G1/S transition (K-Ras, p16 and p53, etc.) [90], thereby making these cells more reliant on the G2 checkpoint for radioprotection. Our data show that the inhibition of Rac1 abrogates the IR-induced G2 checkpoint activation in the pancreatic cancer cells (see Fig. 3–6) but only has subtle, if any, effect on the IR-induced G1 and G2 checkpoint responses of the normal HPNE cells (see Fig. 3D). Additional experiments done in vivo using mouse models will be needed to assess the selectivity of Rac1 inhibitors and identify the mechanisms responsible for this selectively.

Radiation therapy is a staple cancer treatment approach, but its efficacy is still limited by the intrinsic radioresistance of pancreatic cancer cells. Radiation impedes cancer cell growth by inducing cytotoxicity, mainly caused by DNA damage. However, radiation can also simultaneously induce multiple signaling pathways that promote cell survival, such as those mediated by AKT, ATM/ATR and ERK. The pro-survival signaling pathways generally lead to suppression of apoptosis, activation of cell cycle checkpoint and initiation of DNA repair. These signaling pathways act conjointly to reduce the magnitude of radiation-induced cytotoxicity and promote radioresistance in cancer cells. Results in this report provide evidence supporting a novel function for Rac1 in the survival of pancreatic cancer cells after IR, which include the roles of Rac1 in the activation of G2/M checkpoint response and in the suppression of apoptosis induction following IR. Thus, a better understanding of the mechanisms that promote survival following IR would potentially allow for the identification of novel therapeutic targets to be explored for radiosensitization of pancreatic cancer cells.

## MATERIALS AND METHODS

### Cell culture and treatment

Human pancreatic cancer cell lines CD18/HPAF, AsPC-1, Capan-1 and L3.6pl were obtained from American Type Culture Collection (Manassas, VA) and maintained in Dulbecco's Modified Eagle's medium containing 10% fetal bovine serum. HPNE cells are primary human pancreatic ductal cells immortalized using hTERT, the catalytic subunit of human telomerase [64]. HPNE cells were maintained in Medium D medium, which contains 3 parts of high glucose DMEM (Life Technologies, Carlsbad, CA), 1 part of M3F (INCELL, San Antonio, TX), 5% fetal bovine serum and 100 ng/ml recombinant EGF(Life Technologies [64].

Rac1 specific inhibitor NSC23766 [68], was obtained from Tocris Biosciences (Ellisville, MO) and dissolved in water. For experiments involving IR exposure, exponentially growing cells were treated with IR and then incubated at 37^o^C for the indicated time prior to analysis. For experiments involving treatment with both NSC23766 and IR, cells were incubated with NSC23766 for 1 hour prior to IR exposure.

### Antibodies and recombinant proteins

All antibodies were obtained from Santa Cruz Biotechnology (Santa Cruz, CA) unless otherwise indicated. These included mouse IgG for ATM (2C1) (Novus Biologicals, Littleton, CO), Cdc2 (17), Chk1 (G-4), Chk2 (B-4) and poly(ADP-ribose) (3H2844); rabbit IgG for ATM (Ab-3) (EMD Biosciences, San Jose, CA), Caspase 3 (Cell Signaling, Danvers, MA), Cdc2 (C-19), Chk1 (FL-476), Chk2 (Cell Signaling, Danvers, MA), GAPDH (FL-335), Rac1 (C-14); and goat IgG for Actin (I-19), ATR (N-19) and phospho-Cdc2 (Tyr15).

Recombinant PAK-1 protein for Rac1 activity assay was obtained from Addgene (Cambridge, NH) as a glutathione S-transferase (GST) fusion protein containing the full-length human PAK1 protein. Recombinant p53 protein for ATM and ATR kinase assays was a glutathione S-transferase (GST) fusion protein containing full-length human p53 (Addgene, Cambridge, MA). Recombinant Cdc25C protein, the substrate for Chk1 and Chk2 kinase assay, was a GST fusion protein containing residues 200–256 of human Cdc25C (kindly provided by Dr. Helen Piwnica-Worms, Washington University School of Medicine). All GST fusion proteins were purified as described previously [41]. GST was used as a control substrate in all kinase assays and was prepared according to standard procedures (GE Healthcare Bio-Sciences, Piscataway, NJ).

### Immunoblotting, immunoprecipitation and kinase assay

Immunoblotting, immunoprecipitation and kinase assays were performed as described previously [41, 91, 92]. Specific protein signals on Western blots were visualized by chemiluminescence exposed to x-ray film, scanned using EPSON Perfection 4490PHOTO scanner and analyzed using the ImageJ analytical program (NIH, Bethesda, MD).

### Rac1 activity assay

Rac1 activity was assayed using a Rac1 assay kit (Upstate Biotechnology, Lake Placid, NY), as described previously [93, 94]. Briefly, cells were lysed at 4ºC in 25 mM HEPES buffer (pH 7.4) containing 10 mM MgCl_2_, 150 mM NaCL, 1% NP-40, 1 mM EDTA, 2% glycerol, 1 mM DTT, 1 μg/ml aprotinin, 1 μg/ml leupeptin, 1 μg/ml pepstatin, 1 mM phenylmethylsulfonyl fluoride, 1mM sodium fluoride, and 1 mM sodium vanadate. Cell lysates were incubated with agarose beads coated with the GST-PAK1 fusion protein for 1 h to capture GTP-bound Rac1. The obtained GTP-bound Rac1 (Rac1-GTP) was resolved on a 4–20% SDS-PAGE and assessed by immunoblotting using an anti-Rac1 antibody, as described by the manufacturer's instruction. As a negative control, AsPC1 cell lysates were incubated with 1 mM GDP at 30^o^C for 15 min and analyzed for Rac1 activity as instructed by the manufacturer.

### Cell cycle analysis

Fluorescence-activated cell sorting (FACS) analysis was performed on 20,000 cells using a FACS Calibur instrument (Beckon Dickinson, Mansfield, MA), as described previously [41].

### Analysis for mitotic cells

Cells were exposed to IR in the presence/absence of Rac1 specific inhibitor NSC23766, harvested at the indicated times, fixed in 70% ethanol and stained with propidium iodide (PI) and anti-phospho-histone H3 antibody (Upstate Biotechnology, Lake Placid, NY) [37]. Mitotic cells, which contain both *4N*-DNA content and phospho-histone H3 [37], were determined using a FACSCalibur instrument (Beckon Dickinson) and analyzed by using CELLQUEST software. Each analysis was performed using 20,000 cells.

### Adenoviral vectors and adenoviral infections

Recombinant adenovirus N17Rac1 (Ad.N17Rac1) and control adenovirus dl312 (Ad.Control) were kindly provided by Dr. Toren Finkel (NIH, Bethesda, MD). In Ad.N17Rac1, the Rac1 cDNA contains a Ser to Asn substitution at position 17 and functions as a dominant negative mutant [95].

Log-phase cells were infected with either Ad.N17Rac1 or Ad.Control at the indicated doses for 24 h prior to exposure to IR, as described previously [96]. For studies involving Chk1 and Chk2 kinase activity analysis, the irradiated cells were incubated for 1 h post IR and analyzed for Chk1 and Chk2 activities. For studies involving cell cycle analysis, the cells were incubated for 24 h post IR and analyzed for DNA content by flow cytometry [41]. For studies involving mitotic cell analysis, the irradiated cells were incubated for 2 h and analyzed for cells containing both *4N*-DNA content and histone H3-Ser10 phosphorylation [37].

### Clonogenic survival assay

Clonogenic assay was performed as described previously [97]. Briefly, in the presence of no drug or NSC23766, cells were exposed to IR at the doses indicated and incubated for 3 h following IR. The cells were then rinsed with DMEM, re-seeded at the cell number indicated in duplicate and incubated for 10–14 days until colonies formed. The colonies were visualized by crystal violet staining and quantified using ImageJ software as described previously [98].

## SUPPLEMENTARY FIGURE


