# Correction: Inhibition of RAC1 GTPase sensitizes pancreatic cancer cells to γ-irradiation

**DOI:** 10.18632/oncotarget.27450

**Published:** 2020-01-21

**Authors:** Ying Yan, Ashley L. Hein, Asserewou Etekpo, Katrina M. Burchett, Chi Lin, Charles A. Enke, Surinder K. Batra, Kenneth H. Cowan, Michel M. Ouellette

**Affiliations:** ^1^ Department of Radiation Oncology, University of Nebraska Medical Center, Omaha, Nebraska, United States of America; ^2^ Eppley Institute for Research in Cancer and Allied Diseases, University of Nebraska Medical Center, Omaha, Nebraska, United States of America; ^3^ Department of Biochemistry and Molecular Biology, University of Nebraska Medical Center, Omaha, Nebraska, United States of America

**This article has been corrected:** The authors wish to make the readers aware of an error in the actin blot of Fig. 5B. Actin levels were measured once in the six cell extracts used as input for the immunoprecipitations performed in Figs. 5A and 5B. Results of the actin blot were then added to both Figs. 5A and 5B. However, when the actin results were copied into Fig. 5B, lanes were inadvertently shifted by one well. In the revised figure 5B, shown below, the shifted actin blot is deleted. The correct actin blot for both Figs. 5A and 5B is the one shown in Fig. 5A. The authors declare that these corrections do not change the results or conclusions of this paper and apologize for any inconvenience caused by the error.

Original article: Oncotarget. 2014; 5:10251–10270. 10251-10270. https://doi.org/10.18632/oncotarget.2500

**Figure 5:**
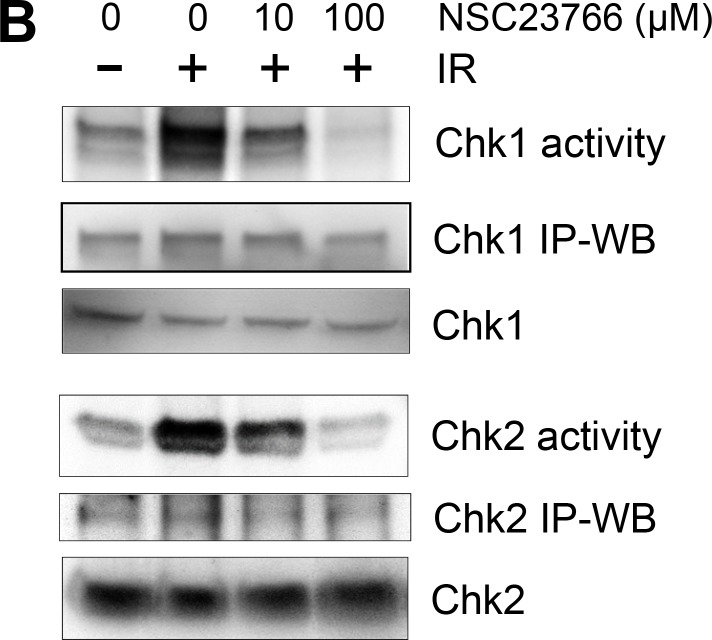
Rac1 inhibition abolishes IR-induced activation of both ATM and ATR signaling pathways. (**B**) To measure Chk1 and Chk2 activity, Chk1 and Chk2 were immunoprecipitated from the cell lysates using anti-Chk1 (G-4) and anti-Chk2 (B-4) antibodies respectively and assayed for relative kinase activity using recombinant Cdc25C protein as substrate. As controls, protein levels of ATR, ATM, Chk1 and Chk2 in the immunoprecipitates (IP-WB) as well as in the cell lysates (WB) were assessed by immunoblotting.

