# Supplementary material for: Inhibition of RAC1 GTPase sensitizes pancreatic cancer cells to γ-irradiation
Source: Oncotarget. 2014 Oct 21;5(21):10251–70. doi: 10.18632/oncotarget.2500 (PMC4279370; doi:10.18632/oncotarget.2500)
Supplement: Supplementary file 1 [file oncotarget-05-10251-s001.pdf]

SUPPLEMENTARY FIGURE

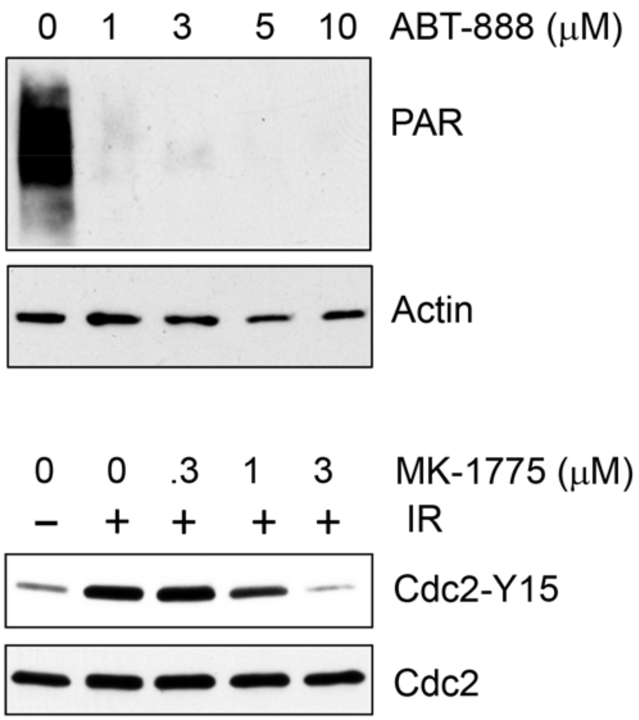

Supplementary Figure S1: Effect of PARP1/2 inhibitor ABT-888 and Wee1 inhibitor MK–1775. Upper panel: AsPC-1 cells were incubated with ABT-888 for 24 h and analyzed for protein poly(ADP-ribosyl)ation by Western blot analysis using an anti-poly(ADP-ribose) antibody. Lower panel: AsPC-1 cells were incubated with MK-1775 at the indicated doses for 1 h, exposed to 10 Gy IR and incubated for additional 3 h. Cdc2 was immunoprecipitated from cell lysates and analyzed for Cdc2-Y15 phosphorylation as described in *MATERIALS AND METHODS*.
